# Structural Insights into the Interaction of Human ALOX15 with the Natural Antioxidant Nordihydroguaiaretic Acid: Functional Inhibitor Studies and Molecular Dynamics Simulations

**DOI:** 10.3390/antiox15030355

**Published:** 2026-03-11

**Authors:** Sonam Grewal, Biswayan Ghosh, Sabine Stehling, Astrid Borchert, Polamarasetty Aparoy, Hartmut Kuhn

**Affiliations:** 1 Molecular Modeling and Protein Engineering Lab, Biology Division, Indian Institute of Petroleum and Energy, Visakhapatnam 530003, Andhra Pradesh, India; sonamgrewal.bio@iipe.ac.in (S.G.); biswayanghosh@iipe.ac.in (B.G.); 2 Department of Biochemistry, Charité-Universitätsmedizin Berlin, Corporate Member of Freie Universität Berlin and Humboldt Universität zu Berlin, Charitéplatz 1, D-10117 Berlin, Germany; sabine.stehling@charite.de (S.S.); astrid.borchert@charite.de (A.B.)

**Keywords:** eicosanoids, ferroptosis, inflammation, atherosclerosis, lipid peroxidation, oxidative stress

## Abstract

Mammalian arachidonic acid lipoxygenases (ALOXs) are lipid-peroxidizing enzymes, which have been implicated in inflammatory, hyperproliferative and neurodegenerative diseases. Nordihydroguaiaretic acid (NDGA) is a naturally occurring antioxidant and a potent lipoxygenase inhibitor. Unfortunately, the molecular basis of the NDGA–ALOX interaction remains unexplored. Here, we show by in silico docking studies and by molecular dynamics simulations that NDGA binds in the substrate binding pocket of human ALOX15 and that Gln595 plays a major role in this interaction. In silico mutagenesis studies (Glu595Ala, Glu595Leu, Glu595Glu, Glu595Ile) modified the stability of the ALOX15–NDGA complex and altered the ligand binding behavior of the enzyme. To validate the in silico findings, we expressed human ALOX15 and the enzyme mutants as recombinant proteins, characterized their functional properties and quantified the IC_50_ values for NDGA-induced inhibition. Consistent with our in silico predictions, the experimental IC_50_ values demonstrated that NDGA strongly inhibited wildtype ALOX15 and its Gln595Glu and Gln595Ile mutants. In contrast, the IC_50_ values for the Gln595Ala and Gln595Leu mutants were more than one order of magnitude higher. These findings highlight the role of Gln595 for the NDGA–ALOX15 interaction and may facilitate the future development of isoform-specific ALOX15 inhibitors.

## 1. Introduction

Lipoxygenases form a diverse family of non-heme iron-containing lipid-peroxidizing enzymes [1,2] catalyzing the stereoselective insertion of molecular oxygen into polyunsaturated fatty acids (PUFAs). The resulting hydroperoxy PUFAs serve as precursors for various mediators (leukotrienes, lipoxins, hepoxilins, eoxins, resolvins) that play important roles in homeostasis and pathology [3,4]. ALOX isoforms frequently occur in highly developed eukaryotes and are rarely present in bacteria [5,6] but have not been detected yet in archaea [6] and viruses [7]. In the human genome, six functional ALOX genes (*ALOX5*, *ALOX15*, *ALOX15B*, *ALOX12*, *ALOX12B*, *ALOXE3*) have been detected [8] and each of them encodes for a functional ALOX isoform (ALOX5, ALOX15, ALOX15B, ALOX12, ALOX12B, ALOXE3). For each human *ALOX* gene an orthologous *Alox* gene (*Alox5*, *Alox15*, *Alox15b*, *Alox12*, *Alox12b*, *Aloxe3*) exists in the mouse reference genome but in addition a functional *Aloxe12* gene was identified [8]. Among the human ALOX isoforms, ALOX15 plays a central role in ferroptosis, inflammation and in the immune response [9,10,11].

In humans, ALOX15 is constitutively expressed at high levels in reticulocytes, eosinophils and airway epithelial cells [12] but also in alternatively activated macrophages [13]. The enzyme oxidizes arachidonic acid (AA) and linoleic acid (LA) to the n-6 hydroperoxy derivatives (15S-HpETE, 13S-HpODE) [14,15], which are rapidly reduced in cellular systems to the corresponding hydroxy compounds (15S-HETE, 13S-HODE). 15-HETE functions as a pro-inflammatory lipid mediator [16] and 13-HODE inhibits cell proliferation and may induce apoptosis [17]. In humans, altered expression of ALOX15 has been associated with diverse pathological conditions, such as atherosclerosis, neurodegeneration, stroke, diabetes, asthma and acute pancreatitis [9,10,11].

Natural and synthetic compounds have been identified as inhibitors of human ALOX15, although only a few have progressed beyond preclinical trials. Synthetic inhibitors include tryptamine sulfonamides [18], 6-benzyloxysalicylates [19], ML351 [20] and utreloxastat [21]. Several naturally occurring compounds, such as baicalein [22], coumarin [23], resveratrol [24], rutin [25], alliin [26] and NDGA [27] have also been reported to inhibit human ALOX15. Since many of these compounds also function as antioxidants, it remains unclear whether their biological effects are related to their antioxidative properties or to their inhibitory effects on ALOX15. Unfortunately, in many studies the ALOX15 inhibitory activities of given compounds have not been tested under in vivo conditions and, thus, the conclusions drawn may be misleading [28].

**Figure 1:**
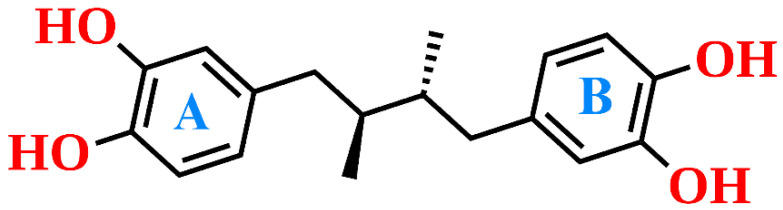
Chemical structure of nordihydroguaiaretic acid (NDGA)**.** NDGA consists of the catechol moieties (cat-A and cat-B) that are interconnected by a branched alkyl chain. The catecholic OH-groups are essential for ALOX inhibition.

Among these inhibitors, NDGA, commercially known as Masoprocol, was selected for this study as it is one of the well-established and extensively characterized inhibitors of human ALOX15. Its ALOX inhibitory activity has been reported in several studies and it exhibits anti-viral, anti-inflammatory and anti-tumor activities [29]. Chemically, NDGA constitutes a phenolic lignan that can be isolated in large quantities from *Larrea tridentata* (creosote bush). It carries two redox-active catechol motifs (Figure 1) and inhibits many ALOX isoforms [30]. Its mechanism of action has been studied and two alternative scenarios have been suggested: (i) it reduces the functional ferric enzyme (ALOX-Fe^3+^) to its catalytically silent ferrous form (ALOX15-Fe^2+^); (ii) it complexes the catalytically active non-heme iron preventing its valency change. These findings prompted a number of follow-up studies aimed at optimizing the inhibitor structure. In this context, Eads et al. explored the bioactivity of terameprocol (M4N or EM-1421), in which the catecholic hydroxyl groups were substituted by methoxy groups [31]. Although the compound lacked any sizable ALOX inhibitory activity, it remained of pharmacological relevance and advanced into clinical phase II trials because of its potent inhibitory activity of prostaglandin synthase 2 (PTGS2) [31,32]. In 2002, Whitman et al. conducted a systematic study in which a series of NDGA analogs were synthesized. From the inhibitor data the authors concluded that the phenolic hydroxyl groups are essential for maintaining ALOX inhibitory activity [33]. These structure–activity relationships suggested that NDGA may bind at the active site of the enzyme and may interact with the enzyme-bound non-heme iron. However, despite these insights, the structural basis for the ALOX15–NDGA interaction has not been explored and it remains unclear at which active site amino acids may directly interact with the inhibitor.

To fill this gap, we first performed in silico docking studies and molecular dynamics (MD) simulations to identify key amino acids contributing to the binding of NDGA in the substrate binding pocket of human ALOX15. Next, we performed in silico and in vitro mutagenesis studies to explore the impact of targeted amino acid exchanges on the stability of the enzyme–inhibitor complex. Taken together, our data provide a deeper insight into the binding of NDGA at the active site of h-ALOX15 and may be of relevance for the development and structural optimization of isoform-specific ALOX inhibitors.

## 2. Materials and Methods

### 2.1. Chemicals

The chemicals utilized in our experiments were obtained from the following sources: AA and authentic HPLC standards of HETE isomers (5*S/R*-HETE, 5*S*-HETE, 8*S/R*-HETE, 8*S*-HETE, 12*S/R*-HETE, 12*S*-HETE, 15*S/R*-HETE, 15*S*-HETE) from Cayman Chem. (Ann Arbor, MI, USA); DPBS from PAN Biotech (Aidenbach, Germany); acetic acid from Carl Roth GmbH (Karlsruhe, Germany); sodium borohydride from Life Technologies, Inc. (Eggenstein, Germany); isopropyl-β-thiogalactopyranoside (IPTG) from Carl Roth GmbH (Karlsruhe, Germany); restriction enzymes from ThermoFisher (Schwerte, Germany); the *E. coli* strain Rosetta2 DE3 pLysS from Novagen (Merck-Millipore, Darmstadt, Germany). Oligonucleotides were synthesized by BioTez Berlin Buch GmbH (Berlin, Germany). Nucleic acid sequencing was performed at Eurofins MWG Operon (Ebersberg, Germany). HPLC grade solvents were purchased from Fisher Scientific (Portsmouth, NH, USA).

### 2.2. Homology Modeling of the Protein Structure of Human ALOX15

The crystal structure of human ALOX15 has not been solved experimentally. However, since human and rabbit ALOX15 orthologs share a high (>85%) degree of amino acid conservation, the X-ray coordinates of rabbit ALOX15 were used to model the 3D structure of the human enzyme [34]. The FASTA sequence of human ALOX15 was retrieved from the UniProt database (UniProt Accession No.: P16050) and the structure was modeled using the Prime module of the Schrödinger Maestro Suite (release 2023-3) employing the X-ray coordinates of rabbit ALOX15 (PDB ID: 2P0M, chain B) as template. A knowledge-based model was developed while keeping the iron ion and the active site ligand (RS075091) in place. In addition, a water molecule was introduced that completed octahedral iron ligand sphere as suggested for human ALOX15B [35]. The generated model was refined by side chain prediction and loop modeling. To reduce unfavorable contacts, energy minimization was performed for 250 picoseconds (ps). MD simulations were carried out for 2 nanoseconds (ns) using the Desmond program (2023-3). To optimize the final protein structure the OPLS_2005 force field [36] was employed. The last frame of the MD simulation was used for molecular docking studies and for in silico mutagenesis experiments.

### 2.3. Ligand Preparation

The 3D structure of NDGA was retrieved from the ChEMBL database [37] and subsequently subjected to geometry optimization and energy minimization using the OPLS_2005 force field. Protonation states were assigned with the Epik program (2023-3) [38] of the Schrödinger suite. The optimized structure was extracted and further utilized for molecular docking experiments.

### 2.4. Molecular Docking

Molecular docking was carried out using the GOLD software (2024.1.0) [39], which employs a genetic algorithm to predict NDGA binding conformations within the active site of wildtype h-ALOX15. The substrate binding pocket was defined by generating a 6 Å radius around the centroid of the active site ligand (RS075091). Docking was performed with the ChemPLP scoring function using its default settings and histidine protonation states were assigned by the Hermes visualizer. The iron coordinating water molecule that was introduced during structural modeling was extracted for the docking studies. Ten docking conformations were generated, with early termination enabled when the top three poses achieved an RMSD ≤ 1.5 Å. The genetic algorithm was configured with a population size of 100, niche size of 2, 100,000 operations, five islands, a selection pressure of 1.1, and default operator weights of 95, 95, and 10 for crossover, mutation, and migration, respectively. Cut-off distances of 3.5 Å for hydrogen bonds and 4.0 Å for van der Waals interactions were applied. The top-ranking pose was further subjected to interaction analysis and MD simulation.

### 2.5. Molecular Dynamics (MD) Simulation of Wildtype h-ALOX15–NDGA Complex

MD simulations were carried out for the protein–ligand complex using the Desmond (2023-3) to characterize the dynamic behavior of the complex, to analyze its structural stability and to visualize the conformational alterations over time. The system was prepared using the OPLS_2005 force field and was solvated in TIP3P water molecules [36] within an orthorhombic box. Further, the system was neutralized by adding Na^+^ and Cl^-^ counter ions, with positional restraints applied to ensure that no ion was placed within a 20 Å radius around the ligand. Energy minimization was carried out for 250 ps to remove unfavorable interactions. Under an NPT ensemble, equilibration was carried out using a Martyna–Tobias–Klein barostat and a Nosé-Hoover thermostat to maintain 1.01325 atmospheric pressure and 300 K, respectively. A 500 ns production run was executed and trajectory frames were recorded at 500 ps intervals.

### 2.6. In Silico Mutagenesis Studies

From in silico docking studies, a number of amino acid residues were identified that directly interacted with the ligand (NDGA) within the active site of h-ALOX15. The important amino acid forming consistent interaction with NDGA had been identified. Further, to test their relevance for protein–ligand interactions, we performed in silico amino acid substitutions using the PyMOL program(2.5.8). Docking and MD simulations were performed using the same protocol established for the wildtype human ALOX15–NDGA complex. The molecular interactions of the wildtype enzyme and of the various mutant complexes were analyzed using the Biovia Discovery Studio (v24.1.0.23298). The interatomic distances between the key protein residues and the ligand were measured to study the potential non-bonded interactions (hydrogen bonds, π–π stacking, hydrophobic contacts) to understand the binding mode and to study the effect of mutagenesis.

### 2.7. Recombinant Expression of Wildtype Human ALOX15 and of Selected Enzyme Mutants

The ALOX15 variants were expressed as N-terminal hexa-his-tag protein following the expression protocol described previously for *P. aeruginosa* ALOX [40]. *E. coli* competent cells (strain Rosetta 2 DE3 pLysS) were transformed with 50–100 ng of the recombinant expression plasmid and were cultured overnight on agar plates containing kanamycin and chloramphenicol. A single bacterial colony was picked, and two 1 mL bacterial liquid cultures (LB medium with 50 μg/mL kanamycin and 35 µg/mL chloramphenicol) were grown for 6–8 h at 37 °C. Measurements of 50 mL culture medium (Enpresso^®^ B kit, Enpresso GmbH, Berlin, Germany) containing kanamycin (50 µg/mL) and chloramphenicol (35 µg/mL) were then added to the pre-cultures and the transformed bacteria were grown overnight in ultra-yield culture flasks (Thomson Instrument Company, Oceanside, CA, USA). Recombinant protein expression was induced by the addition of IPTG (1 mM final concentration) and booster tablet and reagent A were added according to the manufacturer’s instructions (Enpresso^®^ B kit, Enpresso GmbH, Berlin, Germany). Then, the cultures were maintained at 22 °C for 18 h. Bacteria were harvested by centrifugation; the resulting pellet was reconstituted in DPBS reaching a total volume of 5 mL and the cells were lyzed by sonication using a Branson W-250P tip sonifyer (Heineman, Schwäbisch-Gmünd, Germany). Cell debris was removed by centrifugation (15 min, 15,000× *g*, 4 °C); aliquots of the cell-free lysis supernatants were snap-frozen in liquid nitrogen and stored at −40 °C.

### 2.8. In Vitro Site-Directed Mutagenesis Studies

Site-directed mutagenesis was performed using the PfuUltra II Hotstart PCR kit (Agilent Technologies Germany GmbH & Co. KG, Waldbronn, Germany). Briefly, 10–50 ng of recombinant plasmid DNA was incubated with the mutagenesis primers (1 µL of a 5 µM solution) and 12.5 µL Pfu UltraI II Hot Start PCR master mix in a total volume of 25 µL. PCR amplification was carried out using the following protocol: initial denaturation at 95 °C for 1 min, amplification cycle: 30 s at 95 °C (denaturation phase), 60 s at 55 °C (annealing phase), 10 min at 68 °C synthesis phase. The amplification cycles were repeated 18 times. Subsequently, the parent cDNA was digested with 1 µL DpnI (Thermo Scientific, Schwerte, Germany) for 30 min and digestion was stopped by incubating the samples at 80 °C for 10 min. Measurements of 8 µL of the PCR sample were used to transform competent *E. coli* XL-1 Blue cells (Agilent Technologies Inc., Santa Clara, CA, USA). After incubation for 30 min on ice, the cells were heat-shocked for 45 s at 42 °C, kept on ice for two additional min and then 400 µL SOC medium was added. After 1 h incubation at 37 °C, the cells were plated on an LB agar plate supplemented with 50 µg/mL kanamycin and incubated overnight at 37 °C. Four distinct individual bacterial colonies were selected and 1 mL liquid cultures were grown overnight in LB medium. Finally, plasmid DNA was prepared using the NucleoSpin Plasmid kit (Macherey & Nagel, Düren, Germany) and DNA sequencing (Eurofins Genomics Germany GmbH, Ebersberg, Germany) was carried out to confirm nucleotide exchanges.

### 2.9. SDS-PAGE and Quantitative Western Blotting

To quantify the degree of enzyme expression, aliquots (2–20 µL) of the cell-free bacterial lysate supernatants were analyzed by SDS-PAGE on a 7.5% polyacrylamide gel. Separated protein bands were transferred onto a blotting membrane (Serva GmbH, Heidelberg, Germany) by a wet-blotting procedure. The membranes were blocked with blocking solution (Serva GmbH, Heidelberg, Germany), were washed three times with DPBS containing 0.3% TWEEN 20 and were finally incubated with an anti-his-tag HRP-labeled antibody (ThemoFisher, Schwerte, Germany) at room temperature for 1–2 h. After washing, the membrane was stained using the SERVALight Polaris CL HRP WB Substrate kit (SERVA GmbH, Heidelberg, Germany) for 5 min at room temperature. Chemiluminescence was quantified using the FUJIFILM Luminescent Image Analyzer LAS-1000plus (Fujifilm Europe GmbH, Düsseldorf, Germany). For quantification of the luminescence intensity, different amounts of purified *M. fulvus* ALOX [41] were applied to immunoblotting, which was also expressed as N-terminal hexa-his-tag fusion protein.

### 2.10. In Vitro Arachidonic Acid Oxygenase Activity Assay

To quantify the AA oxygenase activities of the different ALOX15 preparations, aliquots of the cellular lysis supernatants (0.5–5 µL) were incubated in 0.5 mL of DPBS containing 50 µM AA as substrate fatty acids for 3 min at room temperature in a 2 mL Eppendorf tube. The ALOX reaction was terminated by the addition of 1 mg solid sodium borohydride, which inactivated the enzyme (reduction to the catalytically silent ferrous form) and reduced the hydroperoxy fatty acids formed during the ALOX reaction to the more stable hydroxy compounds. After a 5 min incubation on ice, 35 µL of glacial acetic acid was added to acidify the sample. Then, the cellular lysate proteins were precipitated by the addition of 0.5 mL of acetonitrile and following a 15 min incubation period on ice the protein precipitate was spun down. The supernatant was recovered and aliquots were analyzed by RP-HPLC for the formation of ALOX products. For control purposes, a no-enzyme control incubation was carried out. For these incubations we used a bacterial lysate supernatant that was prepared from *E. coli* cells, which have been transformed with the non-recombinant expression plasmid. In these control incubations the formation of oxygenated AA derivative was minimal.

### 2.11. RP-HPLC Analysis of Arachidonic Acid Oxygenation Products

To quantify the amounts of hydroxylated AA derivatives, we routinely employed RP-HPLC with UV detection. For this purpose, aliquots of the AA oxygenase activity assays were injected into a Shimadzu HPLC system equipped with a SIL-20AC auto-injector. Analytes were separated isocratically on an EC 250/4 Nucleodur 100-5 C18 column (Macherey-Nagel, Düren, Germany) connected with an EC 4/3 Nucleodur 100-5 C18 ec pre-column (Macherey-Nagel, Düren, Germany) at room temperature using a solvent flow rate of 1 mL/min. A mobile phase consisting of acetonitrile: water: acetic acid (70:30:0.1, by vol.) was used and the UV absorbance at 235 nm was recorded. Complete UV spectra of the analytes were stored every 0.3 s. For quantitative purposes, the intensity scale of the chromatographic device was calibrated by injecting known amounts of 15-HETE and a five-point calibrations curve was established.

### 2.12. Combined Normal Phase/Chiral Phase High Performance Liquid Chromatography (NP/CP-HPLC)

Mono-hydroxylated PUFAs are optically active compounds and they occur in two enantiomers (*R* vs. *S* isomers). Racemic mixtures of *R* and *S* isomers are usually formed via autoxidation, but ALOX products are overwhelmingly chiral. Thus, quantification of the *S/R* ratio of the oxygenated polyenoic fatty acids allows conclusions on the metabolic origin of these compounds. Unfortunately, hydroxy fatty acid enantiomers can neither be separated by conventional RP- nor by NP-HPLC. In fact, for enantiomer separation, HPLC columns with a chiral stationary phase are required. To separate hydroxy polyenoic fatty acid enantiomers, we employed a Daicel (Osaka, Japan) Chiralpak AD-H column (250 mm × 4.6 mm, 5 µm particle size). For this purpose, a mobile phase consisting of n-hexane:methanol:ethanol:acetic acid (96:3:1:0.1, by vol.) was used. For mobile phase preparation, 30 mL of methanol was first mixed with 10 mL of ethanol and 1 mL of acetic acid and then this mixture was added to 960 mL of n-hexane. The mobile phase was sonicated in a Sonorex Super RK512H ultrasonic bath (Bandelin GmbH, Berlin, Germany) and used the same day. The flow rate was set to 1.6 mL/min and the lipid extracts were analyzed at room temperature. The UV absorbance of the column eluate at 235 nm (conjugated dienes) was recorded.

### 2.13. Quantification of the IC_50_ Values

To quantify the NDGA IC_50_ values for wildtype human ALOX15 and the different enzyme mutants, we first prepared the following NDGA solutions (in DMSO) from a 10 mM stock solution (3.02 mg/mL) by serial dilution: 3 mM, 1 mM, 300 µM, 100 µM, 30 µM, 10 µM, 3 µM, 1 µM 0.3 µM, 0.1 µM. A measurement of 2 µL of these solutions was then added to 200 µL of DPBS containing 100 µM AA. After a 10 min equilibration period, the ALOX15 reaction was started by the addition of 5 µL bacterial lysate supernatant. After a 3 min incubation period, the ALOX reaction was terminated by the addition of 1 mg solid sodium borohydride. The samples were incubated on ice for 5 min, and 30 µL of glacial acetic acids and 200 µL of acetonitrile were added. Protein precipitate was spun down and aliquots of the protein-free supernatants were injected to RP-HPLC to quantify the amounts of AA oxygenation products. Two samples without NDGA were run in parallel. The mean of the product formation of these control incubations was set to 100% and dose–response curves were plotted. For quantification of the IC_50_ values, the residual ALOX15 activity was measured once at each inhibitor concentration. Thus, the IC_50_ values for each enzyme species are based on 11 different measuring points covering in an NDGA concentration range of five orders of magnitude.

### 2.14. Sample Repetitions, Statistic Evaluation and Image Preparation

If not indicated otherwise, 3–4 repetitions were run for each sample. The area units of the HPLC peaks were converted into nmoles conjugated dienes after calibration of the chromatographic system (injection of five different amounts of 15-HETE). Statistical evaluation of the experimental raw data and quantification of the patterns of AA oxygenation products was carried out with the two-sided t-test using the GraphPad Prism software package (version 8.3.1). Numeric *p*-values < 0.05 were considered statistically significant. The images were prepared using the GraphPad Prism software (version 8.3.1) and the Adobe Photoshop program (version 21.1.1).

## 3. Results

### 3.1. NDGA Binding at the Active Site of Human ALOX15 and Gln595 Is of Mechanistic Relevance

**Figure 2:**
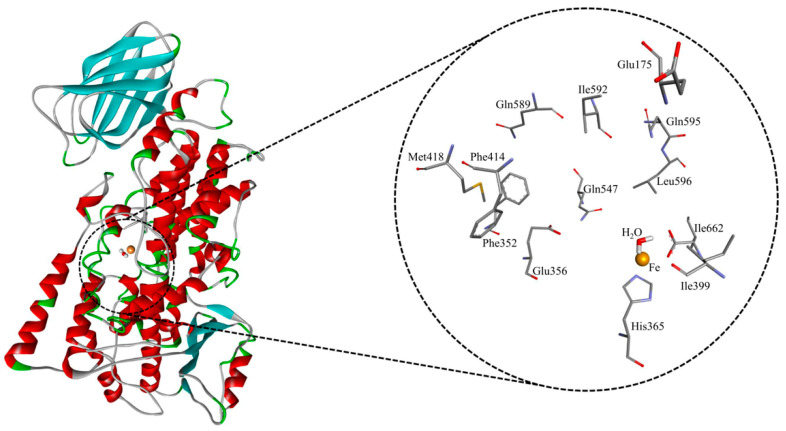
Structural model of human ALOX15 and active site amino acids, which have been implicated in enzyme–substrate interactions. His360, His365 and Ile662 are first order iron coordinating amino acids. Met418, Phe352, and Ile592 are triad determinants, which are important for the reaction specificity of mammalian ALOX15 orthologs. Gln595 fine-tunes the reaction specificity of mammalian ALOX15 orthologs. The other amino acids (Phe414, Gln589, Glu356, Gln547, Leu596, Asp173 and Ile399) also function as constituents of the substrate binding pocket.

To elucidate the molecular basis of NDGA binding within the catalytic pocket of h-ALOX15, molecular docking studies and MD simulations were performed. Initially, the top-ranked docking pose of NDGA was used to identify the surrounding amino acid residues. These residues involved Glu175, Phe352, Glu356, His365, Ile399, Phe414, Met418, Gln547, Gln589, Ile592, Gln595, Leu 596 and Ile662 (Figure 2).

The docking analysis indicated that NDGA interacted with active site amino acids in different ways including hydrogen bonds, hydrophobic contacts, water-mediated hydrogen networks, and π–π electron stacking. Structurally, NDGA consists of two catechol rings, which are connected by an alkyl chain (Figure 1). In the active site, one of the catechol moieties, i.e., cat-A, was positioned close to the catalytic iron forming a hydrogen bond with the backbone atoms of Ile662. The other catechol ring, cat-B, formed two hydrogen bonds with the backbone atoms of Ile592 and the side chain of Gln595. The linker chain of the inhibitor was lined by various hydrophobic residues and was stabilized via π–π and π–alkyl interactions.

### 3.2. The ALOX15–NDGA Complex Is Fairly Stable and Survived the Simulation Period

To assess the stability of the ALOX15–NDGA complex and to characterize the binding dynamics, a 500 ns molecular dynamics simulation was performed (Video S1). For these simulations, the top-ranked docking pose was used as starting conformation. Initially, both catechol rings of the NDGA ligand were involved in hydrogen bonding, one (cat-A) with Ile662 and the other (cat-B) with Ile592 and Gln595. During the simulation period the orientation of the cat-B ring was slightly altered and the entire structural unit moved towards Gln595 to form a stable hydrogen bond. Moreover, we observed a reduction in the distance between the two catecholic hydroxy groups of the cat-B moiety and the site chain of Gln595 (Table 1) from 3.25 Å to 3.05 Å. This distance shortening suggests that Gln595 might play an important role in NDGA binding at the active site of human ALOX15.

**Figure 3:**
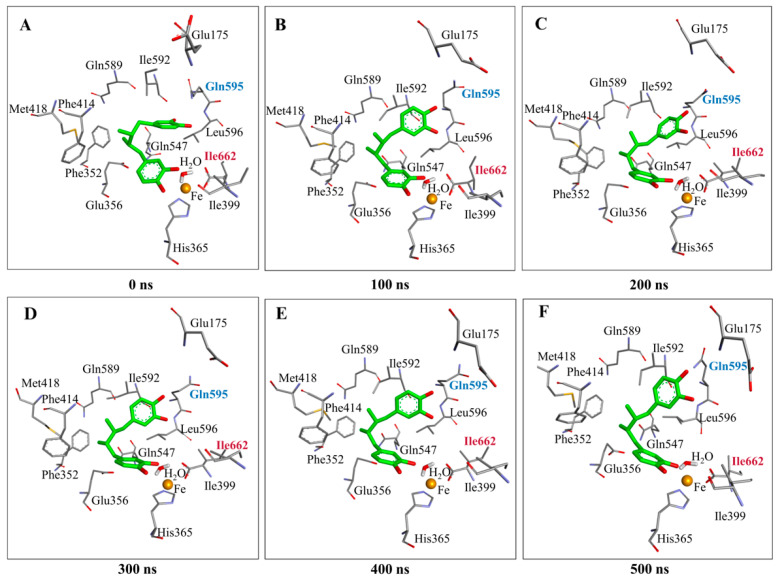
Docking poses of NDGA at the substrate binding pocket of recombinant human ALOX15 at different time points of MD simulation. NDGA was docked into the substrate binding pocket of human ALOX15 and MD simulations were carried out. At different time points of the simulation period the 3D structure of the ALOX15–NDGA complex is visualized, (**A**) 0 ns, (**B**) 100 ns, (**C**) 200 ns, (**D**) 300 ns, (**E**) 400 ns and (**F**) 500 ns. Residues forming hydrogen bonds with cat-A are labeled in maroon, and those interacting with cat-B are labeled in cyan.

Interestingly, the position of the cat-A ring remained unaltered and this structural subunit interacted with backbone atoms of Ile662 (Figure 3). This interaction remained intact throughout the entire simulation period indicating that this enzyme–ligand interaction is rather stable. Gln595 has previously been suggested as one of the key amino acids within the active site of human ALOX15 [42]. It has been implicated in substrate binding and protein stability but may also be important for the allosteric properties of the enzyme. In fact, previous modeling studies and in vitro experiments suggest that Gln596 may not directly interact with the carboxylic group of the fatty acid substrate. In contrast, mutations of Gln596 destabilized the enzyme structure, which may be related to a disturbance in the electrostatic interaction network with other amino acids in the immediate surroundings [42]. Moreover, previous MD simulations on human ALOX15 dimers suggest that the geometry of the inter-monomer interface within the ALOX15 dimer depends on the structure of the substrate fatty acid bound inside the substrate binding pocket of the enzyme and that Gln596Ala exchange modifies the allosteric properties of the enzyme.

### 3.3. In Silico Mutations of Gln595 Destabilized the Structure of Some ALOX15–NDGA Complexes

To characterize the detailed role of Gln595 for NDGA binding, we next performed in silico mutagenesis studies followed by MD simulations for the following human ALOX15 point mutants: (i) Gln595Ala (Video S2), (ii) Gln595Leu (Video S3), (iii) Gln595Ile (Video S4), (iv) Gln595Glu (Video S5). The Gln595Ala exchange was performed to evaluate the impact of reduced side chain volume.

Gln595Leu and Gln595Ile exchanges were more or less volume neutral, but the polarity of the Gln side chains was higher than that of Leu and Ile. Moreover, the aliphatic side chain of Leu is more flexible than that of Ile. Finally, a Gln595Glu exchange was selected to explore the impact of a negative charge on the ALOX15–NDGA interaction.

**Figure 4:**
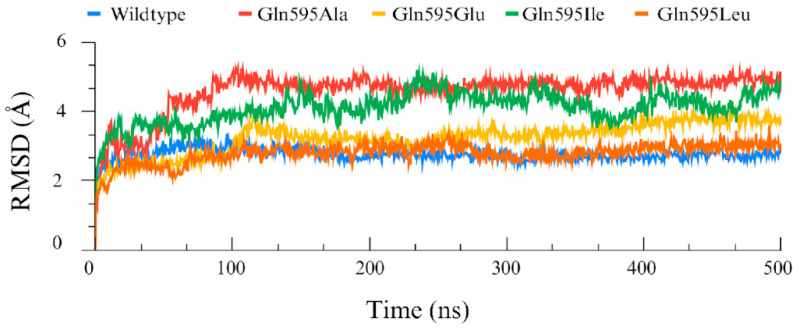
Protein RMSD profiles of wildtype human ALOX15 and of its Gln595 mutants over a 500 ns MD simulation period. Cα-RMSD trajectories for the wildtype (blue), Gln595Ala (red), Gln595Glu (yellow), Gln595Ile (green) and Gln595Leu (orange) shown. The data indicate that after a 100 ns induction period all enzyme–inhibitor complexes have reached a stable configuration.

After creation of the different point mutants, NDGA was docked into the active site of each mutant enzyme and the respective top-ranked docking poses were used for MD simulations. To evaluate the dynamic stability of the ALOX15–NDGA complexes, root mean square deviation (RMSD) analysis was performed. The RMSD profiles of the C-α backbone atoms (Figure 4) indicated that all systems reached convergence after about 100 ns and remained structurally stable over the 500 ns MD simulation.

**Figure 5:**
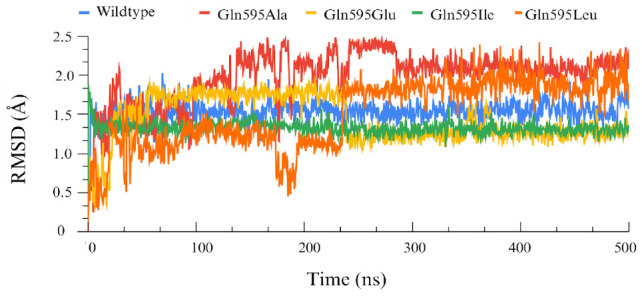
Ligand RMSD profiles of wildtype human ALOX15 and its Gln595 mutants over a 500 ns MD simulation period. Ligand trajectories for the wildtype (blue), Gln595Ala (red), Gln595Glu (yellow), Gln595Ile (green) and Gln595Leu (orange) are shown. This plot indicates that the structure of the ligand was stabilized during the initial simulation period but converges at the end of the simulation.

**Figure 6:**
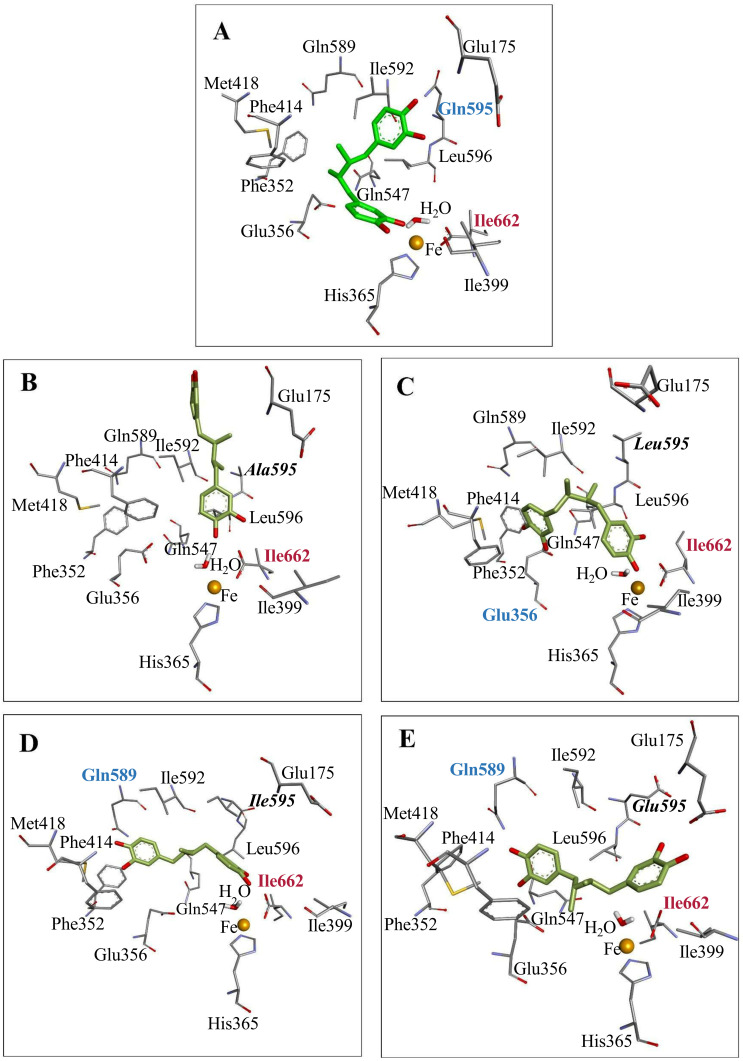
Docking poses of NDGA in the substrate binding pocket of human ALOX15 variants after a 500 ns MD simulation period. MD simulations were carried out as described in Section 2 and the 3D structure of the ALOX15–NDGA complex after the simulation period is shown. (**A**) Wildtype ALOX15 (Gln595), (**B**) Gln595Ala ALOX15, (**C**) Gln595Leu ALOX15, (**D**) Gln595Ile ALOX15, (**E**) Gln595Glu ALOX15. This data indicates that NDGA adopts a different configuration at the active site of the different ALOX15 mutants. Residues forming hydrogen bonds with cat-A are labeled in maroon, and those interacting with cat-B are labeled in cyan.

When a less space filling residue (Gln595Ala) was introduced at position 595 the structure of the ligand (NDGA) was less stable during the first 300 ns of the simulation period (Figure 5). In fact, during the early simulation period the ligand experienced a conformational drift within the active site. However, after 300 ns the ligand adopted a stable conformation in the substrate binding pocket interacting with the backbone atoms of Ile662. A similar interaction was also observed for the wildtype enzyme (Figure 6A). Most interestingly, however, the opposite end of the ligand extended into the void volume that was created by Ala introduction. In fact, the ligand even partly left the substrate binding pocket (Figure 6B). Compared with Gln, the side chain of Leu possesses a smaller hydrophobic side chain, leading to an increase in local volume. The aliphatic side chain of a Leu is much more flexible; thus, it can be pushed in different directions. In MD simulation of the NDGA–ALOX15 (Gln595Leu) complex the ligand attains a stable conformation after a 200 ns simulation period (Figure 5). The ligand position was stabilized by a hydrogen bridge of both catechol rings, i.e., cat-A and cat-B, with the backbone atoms of Ile662 and the side chain of Glu356 respectively (Figure 6C). Moreover, the alkyl linker of NDGA was stabilized by a π–π interaction with Leu596 and π–alkyl interactions with Leu595. Similar to Leu, the side chain of Ile is somewhat less space filling than that of Gln but the gain of space induced by the Gln595Ile mutation (Figure 6D) is limited. The aliphatic side chain of Ile is less flexible when compared with that of Leu. The most important consequence of Gln595Ile exchange is the reduction in polarity at the mutation site. MD simulations with the h-ALOX15 Gln595Ile mutant suggested that the ALOX15–NDGA complex remained relatively stable throughout the simulation period (Figure 5). Molecular interaction analysis showed that for the Gln595Ile mutant the cat-A ring of the ligand maintained a hydrogen bond with Ile662. This interaction was also observed for the wildtype enzymes. The cat-B ring was somewhat dislocated into the direction of Gln589 and Met418 and formed stable hydrogen bonds with these two residues (Figure 6D). In addition, Gln595 formed a π–alkyl interaction with the ligand. Overall, the cat-A moiety remained localized near the Ile662 throughout the simulation and this data suggests a stable position of the ligand in the substrate binding pocket.

Gln and Glu have comparable side chain geometries and, thus, Gln595Glu exchange was not expected to induce major alterations in the active site volume. However, this amino acid exchange introduces a negative charge and, thus, this amino acid exchange was expected to compromise the stability of the ALOX15–NDGA complex. In fact, during the initial phases of our simulation period the complex appeared to be unstable. However, after 200 ns NDGA adopted a stable position in the substrate binding pocket as indicated by the ligand RMSD profile (Figure 5). At the molecular interaction level, simulation analysis revealed that one catechol (cat-A) moiety of NDGA maintained a relatively stable hydrogen bond with Ile662 but, in addition, it established a new hydrogen bond with Gln175. The second catechol (cat-B) ring formed hydrogen bonds with Gln589 and Glu356 (Figure 6E). This interaction pattern suggests that Gln589 might compensate for the defective effect of Gln595Glu exchange on the stability of the ALOX15–NDGA complex. These observations suggested that the Gln595Glu exchange did hardly impact NDGA binding. Moreover, the introduction of a negative charge at position 595 did not adversely affect the stability of the enzyme–ligand complex.

### 3.4. Wildtype ALOX15 and Its Gln595 Mutants Are Well Expressed in E. coli

**Figure 7:**
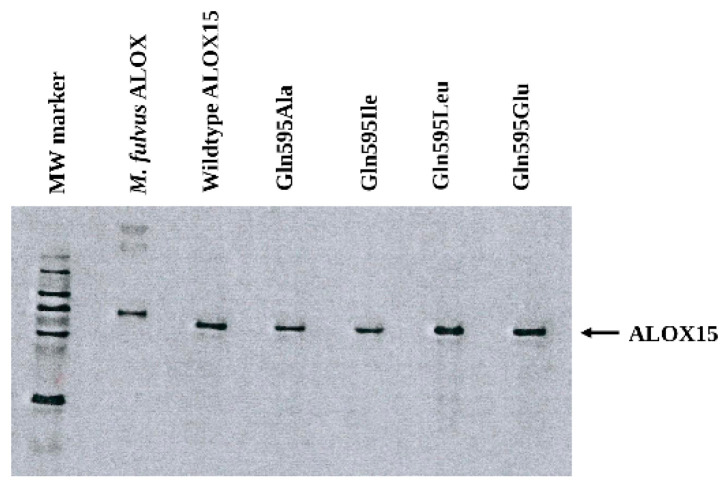
Immunoblot analysis of the recombinant human ALOX15 variants. Human ALOX15 variants were expressed as N-terminal hexa-his-tag fusion proteins in *E. coli* (see Section 2). After disruption of the cells, equal volumes (5 µL) of the bacterial lysis supernatants were applied to SDS-PAGE and the protein blot was stained using an anti-his-tag antibody (see Section 2). The expression levels of the different human ALOX15 mutants are given in Table 2. Lane 1: Molecular weight standards, Lane 2: 1 µg purified recombinant M. fulvus ALOX used as reference protein.

The in silico docking studies and the MD simulations suggested that Gln595 may play a role for NDGA binding at the active site of human ALOX15. To test the functional relevance of this amino acid residue for NDGA-induced inhibition, we next expressed wildtype h-ALOX15 and the corresponding mutants (Gln595Ala, Gln595Ile, Gln595Leu, Gln595Glu) as N-terminal hexa-his-tag fusion proteins in *E. coli* and found that all enzymemutantst were nicely expressed (Figure 7). The expression levels (Table 2), which were estimated by quantitative Western blotting using purified *M. fulvus* ALOX as the reference protein, varied between 30 and 50 mg ALOX protein per liter of bacterial liquid culture.

### 3.5. Functional Characterization of the Different ALOX15 Gln595 Mutants

For functional characterization of the different ALOX15 mutants, we carried out in vitro activity assays and quantified the amounts of AA oxygenation products formed during a 3 min incubation period. From this data and from the amounts of ALOX protein present in the different enzyme preparations we quantified the relative catalytic activities of the different enzyme mutants. Since we did not purify the recombinant proteins, we were not able to perform detailed kinetic studies determining the K_M_ and k_cat_-values. Such measurements have previously been performed for wildtype h-ALOX15 and the Gln595Ala, Gln9595Leu and Gln595Glu mutants [42] and this data indicated that mutagenesis of Gln595 impaired the catalytic efficiency of the enzyme. However, the reaction specificity of AA oxygenation was only slightly modified [42] and this data indicated that AA oxygenation was completely controlled by the mutant enzymes. In the present study, we normalized the catalytic activities measured for the crude enzyme preparations for similar ALOX15 expression levels and found that most enzyme variants exhibited slightly reduced catalytic activities when compared with the wildtype enzyme (Table 2). This data was consistent with the more detailed kinetic studies reported before [42]. Interestingly, the Gln595Ile mutant, which has not been explored before, was 4-fold more active than the wildtype enzyme (Table 2).

**Figure 8:**
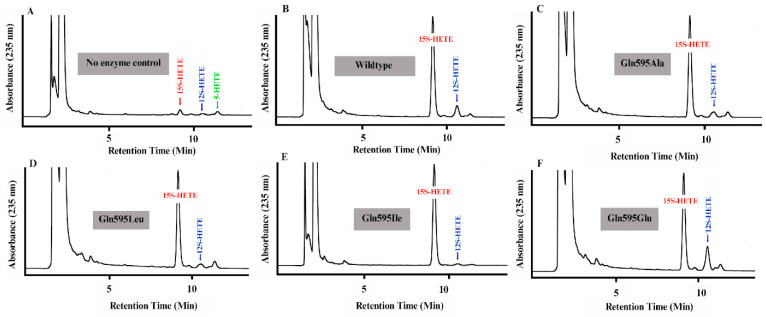
RP-HPLC analyses of the AA oxygenation products formed during the 3 min incubation period of human ALOX15 variants with free AA. In vitro ALOX15 activity assays were carried out with the different human ALOX15 variants as described in the Section 2 and the AA oxygenation products were analyzed by RP-HPLC. The retention time of the authentic standards are indicated above the traces.

Next, we quantified the product patterns formed by the different ALOX15 Gln595 mutants from free AA. When we analyzed the oxygenation products formed during no-enzyme control incubations (Figure 8A, 10 µL DPBS was used instead of bacterial lysate supernatant), only small amounts of conjugated dienes comigrating in RP-HPLC with authentic standards of 12-HETE, 15-HETE and 5-HETE were detected. For wildtype human ALOX15 we confirmed that this enzyme exhibited a dual reaction specificity converting AA to a 10:1 mixture of 15-HETE and 12-HETE (Figure 8B) and this data is consistent with previous reports on the reaction specificity of recombinant human ALOX15 [15,43]. For the Gln595Ala (Figure 8C), Gln595Leu (Figure 8D), Gln595Ile (Figure 8E) and the mutants, the shares of 12-HETE were significantly (Table 2) reduced but for the Gln595Glu (Figure 8F) mutant 12-HETE formation was elevated. Taken together, this data, which is consistent with previous findings [42], suggests that Gln595 is involved in positioning the substrate molecule at the active site of the enzyme. However, the functional alterations introduced by these mutations are rather gradual and we observed neither major functional defects nor pronounced alterations in the reaction specificity of the mutant enzymes. In other words, amino acid exchanges at Gln595 fine-tune the functional enzyme characteristics.

**Figure 9:**
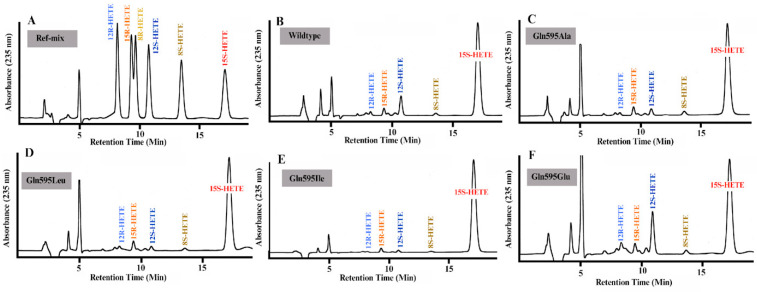
Combined normal phase/chiral phase HPLC analyses of the AA oxygenation products formed during the 3 min incubation period of human ALOX15 variants with free AA. The conjugated dienes formed during the in vitro ALOX15 activity assays by the different ALOX15 variants were prepared by RP-HPLC and further analyzed by combined normal phase/chiral phase HPLC (NP/CP-HPLC) to quantify the enantiomer composition of the major AA oxygenation products. The retention times of the authentic standards are indicated above the traces. (**A**) Chromatogram of the reference mixture. (**B**) Chromatogram of the products formed by wildtype human ALOX15. (**C**) Chromatogram of the products formed by the Gln595Ala mutant of human ALOX15. (**D**) Chromatogram of the products formed by the Gln595Leu mutant of human ALOX15. (**E**) Chromatogram of the products formed by the Gln595Ile mutant of human ALOX15. (**F**) Chromatogram of the products formed by the Gln595Glu mutant of human ALOX15.

Most ALOX isoforms exhibit a pronounce enantio-selectivity and for h-ALOX15 the dominant formation of 15S- and 12S-HETE has previously been shown [14,15]. In contrast, the corresponding R-enantiomers (15R-HETE, 12R-HETE) are only formed in small amounts. Loss in enantioselectivity may be considered a sign of compromised enzyme–substrate interaction. To judge the quality of our enzyme preparations we analyzed the AA oxygenation products formed by the recombinant human ALOX15 variants by combined normal phase/chiral phase HPLC (NP/CP-HPLC) and found a strong preponderance of the 15S- and 12S-HETE isomers over the corresponding R-enantiomers (Figure 9A–F). Taken together, the data shown in Figure 8 and Figure 9 indicate the high quality of the recombinant ALOX preparations.

### 3.6. Gln595Ala and Gln595Leu Exchange Strongly Impaired the Inhibitory Efficacy of NDGA

**Figure 10:**
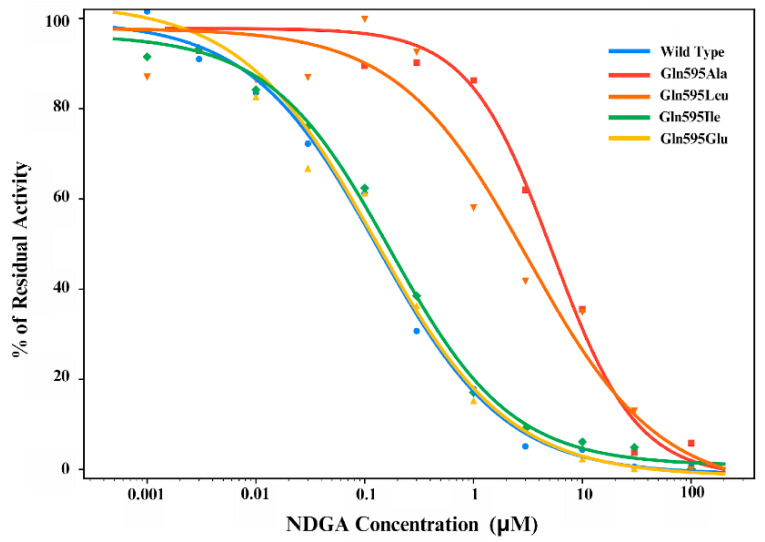
Dose–response curves of ALOX15 inhibition by NDGA for different ALOX15 variants. Catalytic activities of the different human ALOX15 variants were assayed in the presence and absence of different NDGA concentrations as described in the Section 2. Dose–response curves were plotted using the four-parameter logistic (4PL) of non-linear regression [44]. Data fitting was performed in Python (3.12.3) using the curve fit function.

Our in silico mutagenesis studies indicated that mutations of Gln595 alter the position of NDGA in the substrate binding pocket (Figure 6). However, it remained unclear whether these structural alterations may also be related to functional differences in the inhibitory activity of NDGA. To answer this question, we carried out functional inhibitor studies and quantified the dose–response curves of NDGA for the different enzyme mutants. As indicated in Figure 10, wildtype human ALOX15 is strongly inhibited by NDGA as indicated by the IC_50_ value of 126 nM (Table 2). Similar dose–response curves were obtained for the Gln595Ile and the Gln595Glu mutants (Figure 10) and the IC_50_ values were in the same concentration range (Table 2). This result can be attributed to the consistent hydrogen bonding with Ile662 and Gln589, which may function as a prominent replacement for Gln595. Interestingly, the dose–response curves for the Gln595Ala and the Gln595Leu mutants are strongly shifted to higher NDGA concentrations (Figure 10) and the IC_50_ values were 40-fold (Gln595Ala) and 20-fold (Gln595Leu) higher than that of the wildtype enzyme. This data indicates that the Gln595Ala and Gln595Leu exchange strongly impaired the inhibitory potency of NDGA.

To search for the structural reasons for the different inhibitory efficiencies, we performed more detailed MD simulations and found that the system in which the cat-A of the ligand formed a hydrogen bond with Ile662 and the cat-B ring was shifted towards Gln589 displayed IC_50_ values comparable to that of wildtype ALOX15 (Table 2). In other words, Gln589 may stabilize the ALOX15–NDGA when Gln595 is mutated. From this data it can be concluded that Gln595 is not essential for proper NDGA binding. If other amino acids are present at this position, other Gln residues in the immediate surrounding such as Gln589 might stabilize the ALOX15–NDGA complex. In additional experiments we tested whether the presence of NDGA in the activity assay modifies the reaction specificity of AA oxygenation and found that this was not the case. In fact, RP-HPLC analyses revealed similar 15-HETE/12-HETE ratios at different NDGA concentrations. Thus, NDGA modified the catalytic activities of the different enzyme variants but not their reaction specificity.

## 4. Discussion

### 4.1. Degree of Novelty and Advancement of Science

In human ALOX12, His596 has previously been implicated in substrate binding since the positively charged site chain of this amino acid may interact with the negatively charged COO- group of the fatty acid substrate [45]. Similar binding mechanisms have been suggested for mammalian ALOX15 orthologs but in these enzymes the corresponding position is occupied by an uncharged Gln. Despite the missing charge Gln595 may interact with the carboxylate group of the substrate fatty acid via direct H-bonding or indirectly, via a hydrogen bond network of arrested water molecules. Moreover, Gln595 has been implicated in the allosteric regulation of human and rabbit ALOX15 [42]. Together with Ile593, which has been implicated as Triad determinant in the reaction specificity of mammalian ALOX15 orthologs [46,47], it is a constituent of the α-18 helix. In fact, Gln595 is located at the C-terminal end of this helix and thus may play a role in enzyme dimerization [42,48]. Since dimer formation is relevant for the allosteric properties of mammalian ALOX15 orthologs [42,48], this amino acid appears to be of dual functional relevance (substrate binding, allosteric properties). In the present study we tested whether Gln595 may also be important for the binding of other active site ligands such as ALOX15 inhibitors. To start this project, we selected the naturally occurring antioxidant NDGA, which has previously been shown to function as an unspecific ALOX inhibitor. It inhibits most ALOX isoforms at sub-micromolar concentrations [28] and has been suggested to function as a redox-dependent ALOX inhibitor [30]. However, alternative inhibition mechanisms, such as iron chelation, may also be possible. Regardless of the inhibition mechanism, to function as an ALOX inhibitor NDGA must bind in the substrate binding pocket in proximity of the non-heme iron but for the time being it has not been explored how NDGA is bound in the substrate binding pocket of mammalian ALOX isoforms and which amino acids are involved in ligand binding.

To answer these questions, we first performed molecular docking studies of NDGA in the substrate binding pocket of human ALOX15 and found that in the top-ranked docking pose NDGA is bound in proximity of the catalytic non-heme iron. In fact, one of the catechol moieties of NDGA (cat-A) formed a hydrogen bond with the backbone atoms of Ile662. The other catechol ring (cat-B) interacted with the backbone atoms of Ile592 and with the side chain of Gln595. Subsequent MD simulations indicated that this docking pose was fairly stable and multiple side-directed mutagenesis studies in connection with inhibitor assays confirmed the importance of Gln595 for NDGA-induced ALOX15 inhibition. Taken together, our data indicate that Gln595 may not only be of functional relevance for the positioning of fatty acid substrates at the active site of ALOX isoforms and for allosteric regulation of the enzymes but also for the binding of ALOX inhibitors (triple functional relevance).

Gln595 is conserved in mammalian ALOX15 orthologs [42]. However, multiple amino acid sequence alignments of other human and mouse ALOX isoforms (ALOX15, ALOX15B, ALOX12, ALOX12B, ALOX5, ALOXE3) indicated that Gln595 is not strictly conserved. In fact, in human ALOX12 this position is occupied by a positively charged His [45]. On the other hand, Gln589, which is localized in proximity to Gln595 in human ALOX15, showed a higher degree of conservation. This residue may fulfill the functional tasks of Gln595 when this amino acid is mutated. Gln547, which is also localized in proximity to Gln595 and Gln589 in human ALOX15, is strictly conserved in all mouse and human ALOX isoforms and this amino acid is an additional residue that may take over the function of Gln595 in cases that this amino acid is mutated. In other words, in human ALOX15 the functionality of these three Gln residues appears to be redundantly secured. Mutagenesis of one of these amino acids may be without functional consequence since the other residues may step in.

**Figure 11:**
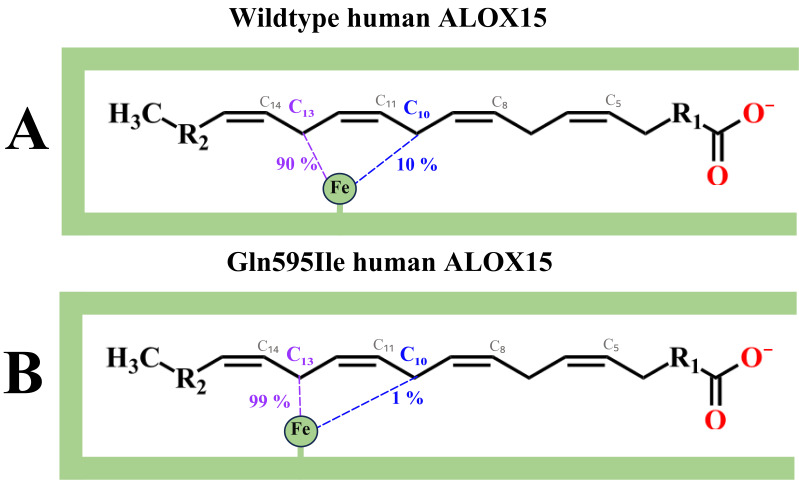
Schematic view of positioning of AA in the substrate binding pocket of wildtype human ALOX15 and its Gln595Ile mutant. (**A**) In wildtype human ALOX15 the enzyme-bound non-heme iron is localized between the carbon atoms C_13_ and C_10_ so that hydrogen abstraction from both bisallylic carbon atoms in a ratio of about 10:1 is possible. (**B**) For the Gln595Ile mutant C_13_ almost exactly aligns with the non-heme iron so that hydrogen abstraction from C_10_ is minimal.

Another novel aspect of our studies is that Gln595Ile exchange induced functional optimization of human ALOX15 (Table 2). In a previous study it has been shown that Gln595Ala, Gln595Leu and Gln595Glu exchange reduced the catalytic efficiency (K_M_/k_cat_ ratio) of h-ALOX15 [45]. Although we did not perform detailed kinetic experiments in the present study, we confirmed this conclusion by our activity assays (Table 2). Interestingly, the catalytic activity of the Gln595Ile mutant was more than four-fold higher than that of the wildtype enzyme and this data suggested that this amino acid exchange did optimize the catalytic efficiency of AA oxygenation. The molecular basis for this optimization has not been explored but it might be possible that the introduction of the less polar Ile may improve binding of the hydrophobic substrate. Alternatively, introduction of the rigid Ile side chain may modify the substrate orientation at the active site so that the bisallylic carbon atom C_13_ of AA gets closer to the non-heme iron (Figure 11). Such a structural arrangement would improve the rate limiting step of the oxygenation reaction. This mechanistic scenario was supported by our specificity data summarized in Table 2. We found that wildtype human ALOX15 converts AA to a 1:10 mixture of 12S- and 15S-HpETE and these findings are consistent with previous reports of the reaction specificity of this enzyme [15,49]. In contrast, the Gln595Ile mutant almost exclusively formed 15S-HpETE since the relative share of 12S-HETE formation was only about 1% (Table 2). This data suggests that in the Gln595Ile mutant the bisallylic carbon atom C_10_ was located more distant from the non-heme iron (Figure 11).

### 4.2. Limitations of the Study

For our inhibitor experiments we routinely performed the in vitro ALOX activity assays with crude recombinant enzyme preparations. Although the catalytic activities of the different enzyme preparations were normalized to a similar ALOX15 protein content, the presence of other proteins may modulate the ALOX15–NDGA interaction. However, since the foreign protein content should be similar in all ALOX15 preparations, the relative differences in inhibitor sensitivity should be reliable. In fact, the two-orders of magnitude differences in the IC_50_ values of wildtype h-ALOX15 and its Gln595Ala and Gln595Leu mutants can hardly be attributed to the presence of foreign proteins. To explore the impact of foreign proteins on the inhibitory efficiency of NDGA, we quantified the degree of inhibition of pure rabbit ALOX15 by 1 µM NDGA in the absence and presence of an ALOX15 deficient bacterial lysate supernatant. Here we found that in the presence of bacterial proteins the degree of inhibition was similar to that in the absence of the foreign proteins. This data indicates that the results of our inhibitor studies are reliable and that the degree of enzyme inhibition is hardly modified by bacterial protein under our assay conditions.

In previous experiments NDGA was identified as a redox inhibitor of mammalian ALOX isoforms [30]. If this hypothesis is correct, ALOX15 catalyzed oxygenation of AA should be inhibited to a similar degree as LA oxygenation. To test this hypothesis, we performed corresponding in vitro inhibitor studies on pure rabbit ALOX15 and found that at 1 µM NDGA LA oxygenation was inhibited to a similar degree as AA oxygenation. Thus, although we did not perform comprehensive dose–response experiments with the two substrates, this data suggests that NDGA is not a substrate-specific ALOX inhibitor and this conclusion is consistent with its activity as a redox inhibitor. However, an iron-chelating mechanism could not completely be ruled out by our data.

Our docking studies and our MD simulations were carried out with a 3D model of human ALOX15, which was based on the crystal structure of the rabbit enzyme ortholog. Unfortunately, the crystal structure of human ALOX15 has not been solved and thus corresponding in silico experiments could not be performed with the X-ray coordinates of the human enzyme. However, since human and rabbit ALOX15 orthologs share a high (>85%) degree of amino acid sequence homology, and since the catalytic properties of the two enzymes are almost indistinguishable, the results obtained in our in silico studies for human ALOX15 should reliably mirror reality.

## 5. Conclusions

This comparative mutational analysis combining computational and experimental approaches highlights the critical role of Gln595 in modulating NDGA binding within the substrate binding pocket of human ALOX15. Furthermore, our data suggested that Gln589 and Gln547 may serve as auxiliary residues contributing to ligand stabilization. These findings provide mechanistic insights into the binding of a potent inhibitor in the substrate binding pocket of mammalian ALOX15 orthologs and are of major relevance for the future development of isoform-specific ALOX15 inhibitors.

## Figures and Tables

**Table 1:** Distance (Å) between the catechol A ring of NDGA and the amino acids Gln589 and Gln595 for different human ALOX15 mutants before (BMD) and after (AMD) the 500 ns MD simulation period.

Enzyme Variants	Distance of Catechol Ring from Gln589 (Å)		Distance of Catechol Ring from Gln595 (Å)	
	Before MD	After MD	Before MD	After MD
WT	7.04	10.6	3.25	3.05
Gln595Ala	13.7	8.9	4.87	7.6
Gln595Glu	3.1	2.92	5.7	9.3
Gln595Ile	4.3	2.7	9.8	6.96
Gln595Leu	3.1	7.7	6.98	5.2

**Table 2:** Expression levels, reaction characteristics and NDGA sensitivity of human ALOX15 mutants. Human ALOX15 variants were expressed as N-terminal hexa-his-tag fusion proteins in *E. coli* as described in Section 2. The cells were lysed by repeated sonication and the 20,000 g supernatants were used as enzyme source. The expression levels of the recombinant proteins were quantified by quantitative immunoblotting using the purified ALOX of *M. fulvus* as reference protein. In vitro activity assays were performed as described in Section 2 and the relative shares of 12-HETE were determined by RP-HPLC. For determination of the IC_50_ values, in vitro activity assays were run in the presence and absence of different concentrations of NDGA ranging between 1 nM and 100 µM. * *p* < 0.05 when compared with wildtype ALOX15 (two-sided *t*-test).

Enzyme Variant	Expression Level (mg ALOX Protein/L Bacterial Liquid Culture)	Relative Specific Catalytic Activity (%)	Relative Share of 12-HETE (%)	IC_50_ for NDGA (nM)
Wildtype	44.8	100 ± 2.0	10.4 ± 0.2	126
Gln595Ile	29.4	443.2 ± 12.1	1.4 ± 0.1 *	159
Gln595Glu	46.2	54.1 ± 3.3	21.6 ± 0.2 *	130
Gln595Ala	35.0	83.2 ± 8.2	4.9 ± 0.2 *	4965
Gln595Leu	49.0	41.4 ± 3.1	1.8 ± 0.1 *	2648

## Data Availability

The original experimental raw data are stored on the servers of the research institutions and are available upon request from the corresponding authors (in silico studies from P.A., in vitro studies from H.K.).

## Supplementary Materials

The following supporting information can be downloaded at https://www.mdpi.com/article/10.3390/antiox15030355/s1, Video S1: h-ALOX15–NDGA wildtype 500 ns MD movie; Video S2: h-ALOX15–NDGA Gln595Ala 500 ns MD movie; Video S3: h-ALOX15–NDGA Gln595Leu 500 ns MD movie; Video S4: h-ALOX15–NDGA Gln595Ile 500 ns MD movie; Video S5: h-ALOX15–NDGA Gln595Glu 500 ns MD movie.

## Abbreviations

The following abbreviations are used in this manuscript: AA, arachidonic acid; LA, linoleic acid; ALOX, arachidonic lipoxygenase; PTGS, prostaglandin synthase; DPBS, Dulbecco phosphate-buffered saline; *E. coli*, *Escherichia coli*; RP-HPLC, reverse phase HPLC; NP/CP-HPLC, combined normal phase/chiral phase HPLC; HPLC, high performance liquid chromatography; IC_50_, half-maximal inhibitory concentration; *M. fulvus*, *Myxococcus fulvus*; MD, molecular dynamics; NDGA, nordihydroguaiaretic acid; PCR, polymerase chain reaction; PUFA, polyunsaturated fatty acid; RMSD, root mean square deviation; SDS-PAGE, sodium dodecyl sulfate polyacrylamide electrophoresis; 13S-HODE, 13S-hydroxy-octadecadienoic acid; 15S-HETE, 15S-hydroxy-eicosatetraenoic acid.
